# The Hall Technique; a randomized controlled clinical trial of a novel method of managing carious primary molars in general dental practice: acceptability of the technique and outcomes at 23 months

**DOI:** 10.1186/1472-6831-7-18

**Published:** 2007-12-20

**Authors:** Nicola P Innes, Dafydd JP Evans, David R Stirrups

**Affiliations:** 1 Dundee Dental Hospital and School, Park Place, Dundee DD1 4HR, UK

## Abstract

**Background:**

Scotland has high levels of untreated dental caries in primary teeth. The Hall Technique is a simplified method of managing carious primary molars using preformed metal crowns (PMCs) cemented with no local anaesthesia, caries removal or tooth preparation. This study compared the acceptability of the Hall Technique for children, their carers, and dentists, and clinical outcomes for the technique, with conventional restorations.

**Methods:**

General dental practice based, split mouth, randomized controlled trial (132 children, aged 3–10). General dental practitioners (GDPs, n = 17) in Tayside, Scotland (dmft 2.7) placed conventional (Control) restorations in carious primary molars, and Hall Technique PMCs on the contralateral molar (matched clinically and radiographically). Dentists ranked the degree of discomfort they felt the child experienced for each procedure; then children, their carers and dentists stated which technique they preferred. The teeth were followed up clinically and radiographically.

**Results:**

128 conventional restorations were placed on 132 control teeth, and 128 PMCs on 132 intervention teeth. Using a 5 point scale, 118 Hall PMCs (89%) were rated as no apparent discomfort up to mild, not significant; for Control restorations the figure was 103 (78%). Significant, unacceptable discomfort was recorded for two Hall PMCs (1.5%) and six Control restorations (4.5%). 77% of children, 83% of carers and 81% of dentists who expressed a preference, preferred the Hall technique, and this was significant (Chi square, p < 0.0001). There were 124 children (94% of the initial sample) with a minimum follow-up of 23 months. The Hall PMCs outperformed the Control restorations:

a) 'Major' failures (signs and symptoms of irreversible pulpal disease): 19 Control restorations (15%); three Hall PMCs (2%) (P < 0.000);

b) 'Minor' failures (loss of restoration, caries progression): 57 Control restorations (46%); six Hall PMCs (5%) (P < 0.000)

c) Pain: 13 Control restorations (11%); two Hall PMCs (2%) (P = 0.003).

**Conclusion:**

The Hall Technique was preferred to conventional restorations by the majority of children, carers and GDPs. After two years, Hall PMCs showed more favourable outcomes for pulpal health and restoration longevity than conventional restorations. The Hall Technique appears to offer an effective treatment option for carious primary molar teeth.

**Trial registration number:**

Current Controlled Trials ISRCTN47267892 – A randomized controlled trial in primary care of a novel method of using preformed metal crowns to manage decay in primary molar teeth: the Hall technique.

## Background

The high level of dental caries in the Scottish child population, with 55% of 5 year old children having visible decay into dentine and 16% having experienced dental extractions [1], imposes a considerable burden on children, their carers, and the dental team looking after them. Many children have to accept toothache as a part of normal daily life [2-4]. Current guidelines recommend combined preventive and restorative management of carious primary teeth [5], yet in Scotland in 1989 only 18% of cavities in 5 year olds had been restored [6], and in 2003 this had fallen to 9% [1]. Alongside the extensive untreated caries, there has been intense debate in the UK on whether restorative care provided in general dental practice is an effective way of managing children with dental caries in primary teeth [4,7]. There is evidence that restorations for primary teeth can be effective in terms of longevity [8-12], but very little of this evidence is derived from the Primary Care setting, where the vast majority of child dental care in the UK takes place. In addition, there is no clear evidence that restorative management of dental caries is associated with a reduction in pain and sepsis experienced by children, although there is a suggestion this may be so [13]. There is also no evidence from the Primary Care setting in the UK supporting one particular restorative technique for primary teeth over another.

Despite preformed metal crowns (PMCs) being recommended as the optimum restoration for managing primary molar teeth where caries involves two or more surfaces [14] and evidence for their effectiveness [15,16], they are not widely used in Scotland, making up only 0.4% of all restorations provided for children in 2001/2 [17]. Investigations into the views of Primary Care dentists in the UK have shown that although there is an apparent appreciation of the research and recommendations supporting their use [18-20], PMCs are not viewed as a realistic treatment option for carious primary molars [18-21]. One study of 93 general dental practitioners (GDPs) [18] found that only 3% used PMCs routinely and 82% never used them. Amongst the barriers to their use reported by dentists, are perceived difficulties with children's ability to accept invasive treatment. There is some evidence supporting this view, with comparisons of children's perception of cavity preparation using rotary instruments against hand instruments showing less discomfort [22,23] and lower physiological and behavioural indicators of stress [24] when rotary instruments were not used. In addition, a randomized controlled trial [23], found that hand excavation without local anaesthesia (LA) gave the least discomfort, and conventional cavity preparation with LA, the most. Furthermore, children requiring additional treatment found the experience of LA even less acceptable at subsequent appointments.

**Figure 1:**
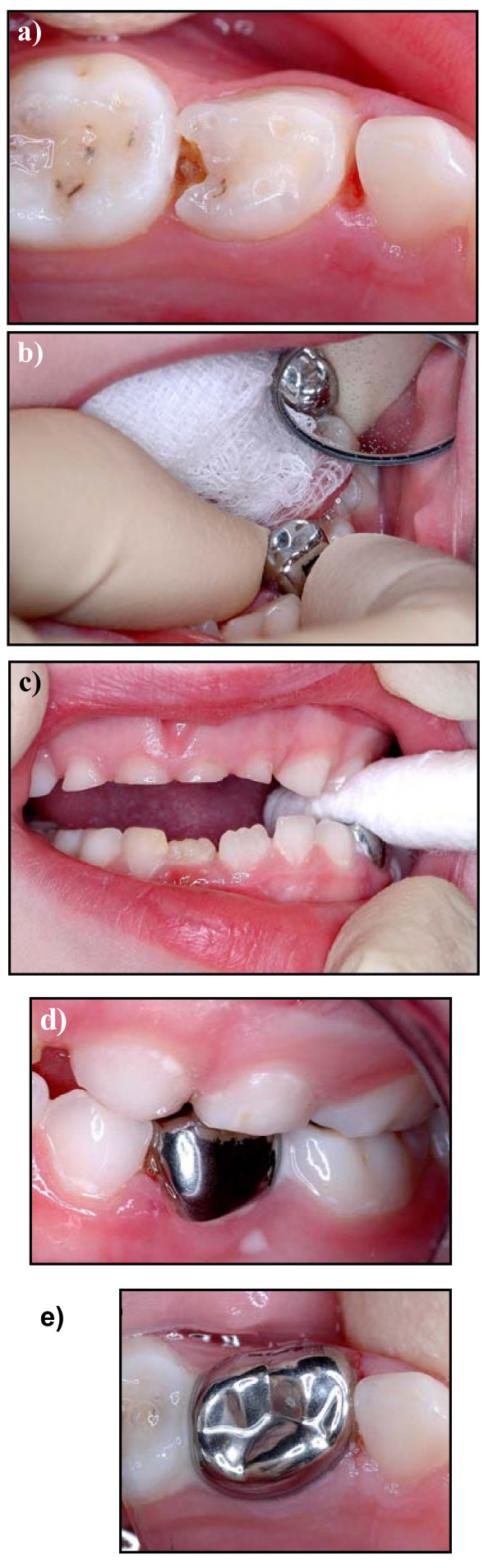
Clinical photographs of a Hall PMC being fitted: a) carious primary molar tooth 74 (LLD) to be fitted with a Hall PMC; b) PMC being tried over occlusal surface of tooth to guage size (guaze providing airway protection). The crown is now filled with glass ionomer cement and placed firmly over the tooth; c) patient biting on cotton roll to push crown between contact points and maintain pressure until cement sets; d) buccal view, and e) occlusal view of the fitted Hall PMC.

It is against this background of low levels of restorative treatment provision in Primary Care, and uncertainty as to the effectiveness of that treatment even if it is provided, that a novel, simplified method of using PMCs, the Hall Technique, has been investigated. This method uses PMCs, which are filled with glass-ionomer cement, and simply pushed onto the tooth with no caries removal, local anaesthesia or tooth preparation (shown in Figure 1). Recently published audit data from Dr Hall's practice records [25] has indicated that the technique (from now on referred to as the Hall Technique) might have similar survival rates to other, more conventional, restorative options currently being used in Primary Care. In addition, avoiding the use of LA and rotary instruments for tooth preparation and caries removal might mean that the technique is less demanding of both children and their dental team, and a pilot trial [26] indicated that it was a technique which children and dentists found acceptable.

The Hall Technique is novel in two ways:

1) the PMC is cemented in place without any tooth preparation or local anaesthesia, and

2) carious tooth tissue is not removed, but sealed into the tooth by the PMC and cement, thus isolating it from the rest of the mouth.

The Hall Technique embraces changing concepts of managing dental caries, moving from the dogma requiring its complete surgical excision, even at the expense of cavity size and pulpal health [27], to the understanding that caries in dentine can be slowed, arrested, and possibly even reversed, within a meticulously sealed environment [28-30].

This study was designed to investigate the effectiveness of the Hall Technique in managing dental caries, and the acceptability of the technique to children, their carers and dentists. It compares conventional restorative methods used by GDPs with the Hall Technique. In order to increase the generalisability of the results, and following the recommendations of the Mant [31] and Clarke [32] reports, the clinical trial was run entirely in general dental practice, as this is where the vast majority of child dental care in the UK is provided.

### Aims

The study had two main aims:

1. to compare the clinical effectiveness of the Hall Technique with conventional methods of managing carious primary molars; and

2. to compare the acceptability of the Hall Technique to children, their carers, and dentists with conventional restorative methods for carious primary molars.

The null hypotheses tested in this paper are that after two years, for restorations placed by GDPs when managing caries in primary molars in this increased caries risk population:

• there is no difference in the incidence of signs and symptoms of pulpal disease between teeth restored with Control restorations and those with PMCs fitted using the Hall Technique;

• there is no difference in longevity of restorations between Control restorations and PMCs fitted using the Hall Technique; and

• children, their carers and GDPs have no preference between the Hall Technique and the conventional restorative care provided by their GDPs.

## Methods

### Study design and ethical approval

The study was a randomized controlled clinical trial, with a split mouth design set in general dental practice in Tayside, Scotland (2000 regional dmft 2.47, d_3 _1.71, mt 0.54, ft 0.22 [33]). Approval from the Tayside Committee on Medical Research Ethics (Ref 108/00) was obtained. Written and verbal consent from parents and, where appropriate, the children, was obtained, following a minimum 24 hours "cooling off" period between their being given information, and consent being obtained.

The GDPs recruited children who had caries affecting pairs of primary molar teeth, which were matched for tooth type, arch and extent of caries. Computer generated randomisation for sequence and side were held centrally, and accessed by telephone to a distant co-ordinator prior to treatment. The tooth on one side was restored using the Hall Technique and the contralateral tooth with the restorative technique the GDP would normally use. Data relating to the two treatments were recorded at the treatment appointment and follow up data at yearly intervals.

### Power calculation

A systematic review of 10 studies [15], comparing the performance of PMCs with amalgam restorations gave a mean failure ratio of 0.32 in favour of the PMCs. Using this data, a two tailed test (α = 0.05), and a power of 80%, it was calculated that a clinical trial would require 58 participants to demonstrate a clinically significant difference in treatment outcomes of 50%. Allowing for loss of participants over a two year period, the target sample size was 120, with a maximum of 200 patients. Sequential analysis was to be undertaken when 90 patients had returned for one year follow-up. If this indicated a significantly increased failure rate for the intervention, recruitment would cease before the maximum of 200 patients.

### Dentists

All 143 GDPs in Tayside, Scotland were invited by letter to participate in the study. From the 41 replies expressing an interest (29% positive response rate), 17 GDPs were selected to obtain a spread of dental practices throughout the region. The GDPs attended a training session at Dundee Dental Hospital, with discussion on the study protocol, training in standardised bitewing radiography techniques using size 0 (22 × 35 mm) films (ULTRASPEED films, Eastman, Kodak, Hertfordshire, UK), Rinn holders (Dentsply, XCP film holding system; Rinn Corp, Elgin, Illinois, USA), and instruction in the Hall Technique.

### Patients

**Figure 2:**
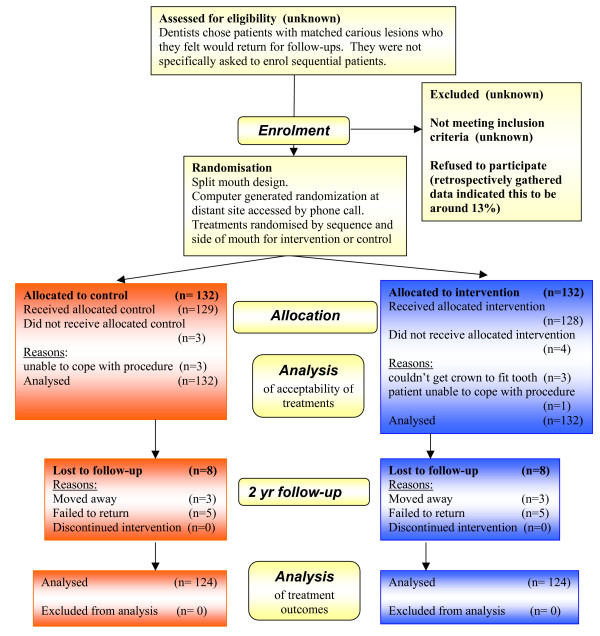
CONSORT (Consolidated Standards of Reporting Trials) diagram showing the flow of participants through each stage of the randomized trial.

Each dentist was asked to recruit 10 patients to the study who had caries affecting pairs of primary molar teeth, which were matched for tooth type, dental arch and extent of caries. Inclusion criteria required children to be 4–9 years of age, with no significant health problem, and presenting for routine dental care to their GDP. Patients had bitewing radiographs taken before entry to the study and then at annual recall visits. Details of patient recruitment and follow-up numbers are listed in the Consort statement (Figure 2).

### Study teeth

For inclusion within the trial, teeth had to be:

• pairs of unrestored carious primary molars;

• matched for tooth type, dental arch, and extent of caries (radiographically ≤ or >1/2 way through dentine); and

• symptomless, with no clinical or radiographic signs of pulpal pathology on bitewing radiography as assessed by the GDP.

**Figure 3:**
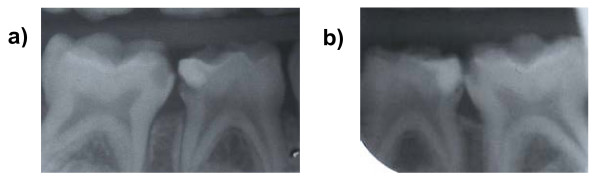
Radiographs of two matched carious lesions in tooth 85 (LRE) – radiograph a) and tooth 75 (LLE) – radiograph b). Patient randomisation number 92.

Where more than one pair of matched carious lesions were present in a child's mouth, the dentist chose which pair should be part of the study. Any carious teeth outwith the study were managed as per the dentists' normal treatment regime. Figure 3 shows the radiographs of a pair of teeth entered into the trial.

### Treatment appointments

The dentists used their discretion to decide whether the two restorations were carried out at the same, or separate appointments, and recorded their decision. The protocol for the provision of the control restoration (Control restoration) followed currently accepted practice, requiring complete removal of caries from the periphery of the cavity, and as far as possible from the base of the cavity without causing pulpal exposure. Local anaesthesia was to be administered if the GDP would usually use it in that clinical situation, and the cavity restored with the material the GDP would normally choose.

The protocol for provision of the Hall Technique PMC (Hall PMC) was as follows:

• obvious food or debris were removed from the cavity but no caries removed;

• the child was positioned upright in the dental chair to reduce the chance of accidental swallowing or aspiration of a loose PMC. Additional airway protection could be gained by use of a gauze swab behind the tooth or by securing the PMC with a strip of elastoplast;

• the correct size of PMC for the tooth was selected i.e. covering all cusps and giving the feeling of "spring-back" when placed up to, but not through, the contact points. If the contacts were very tight, orthodontic separator elastics could be used and the Hall PMC fitted at a subsequent appointment;

• the tooth was rinsed and dried, and the PMC dried;

• the PMC was filled with glass ionomer luting cement. If the cavity was large, some glass ionomer could be placed in the base of the cavity before cementing the crown;

• the PMC was placed evenly over the tooth and the child instructed to bite down firmly until the crown was pushed down over the tooth;

• if the child was unable or unwilling to bite down on the PMC, finger pressure was used to seat the crown;

• extruded cement was removed, and the child asked to keep biting on the Hall PMC until the cement has set; and

• once cement had set, excess cement is removed, floss was used to clear the aproximal contacts, and post-fitting instructions were given.

**Figure 4:**
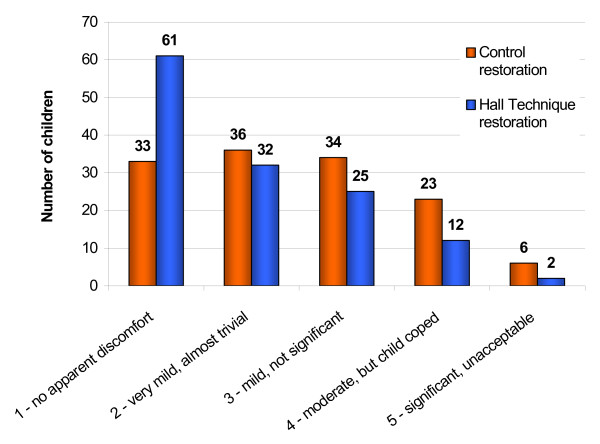
Dentists' estimation of discomfort experienced by child (n = 132 children).

Immediately following each treatment, the GDPs were asked to record the following information on a form:

- any difficulties encountered with either treatment;

- whether caries removal for the control tooth was complete or incomplete (and if incomplete, why);

- the material used for cavity restoration in the control tooth;

- the GDPs subjective assessment of the discomfort level experienced by the child during each procedure, using a five point scale (descriptors listed in Figure 4);

- the distance, measured in millimetres at the incisors, that the Hall crown caused the occlusal vertical dimension (OVD) to be increased by;

- whether the restorations were carried out at the same or separate treatment appointments; and

- the time taken for explanation, and the duration of each treatment.

**Figure 5:**
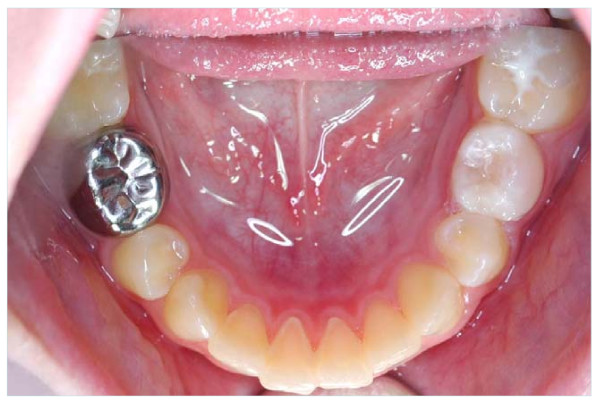
Lower arch with Hall PMC on tooth 85 (LRE) and Control restoration (mesio-occlusal composite) in tooth 75 (LLE). Patient randomisation number 92.

Following recruitment and consent, if either restoration could not be placed, then the patient continued to be included in the trial and monitored as per protocol (following the principle of "intention-to-treat"). On completion of both treatments (see Figure 5), the GDP asked and recorded which treatment option the child, their parent/carer (if present), and they had preferred.

### Patient follow-up

Patients were kept under normal review intervals by their dentists, with clinical and radiographic data (from bitewing radiographs) being recorded at yearly intervals. Details of emergency visits were also recorded. At each annual recall, a pre-printed form was completed, recording:

• a full dental chart;

• whether there was occlusal contact on both sides of the arch;

• whether the child had experienced any temporomandibular joint (TMJ) related pain or difficulty with eating;

• any dental pain the child had experienced and whether the child had required emergency dental treatment. Any emergency treatment required by either of the study teeth outwith routine recall was also recorded; and

• the success or failure of the restorations, assessed clinically and radiographically.

### Success or failure of the restorations

GDPs monitored the success or failure of the restorations using their usual clinical criteria as to whether a restoration required a further intervention, such as repair, replacement or if the tooth required pulp therapy or extraction. If there was a dental intervention, then they recorded this on their pre-printed annual recall form. Two researchers (NI and DE), reviewed these forms, and the GDPs radiographs, and using the criteria in Table 1 ascribed the following outcomes:

**Table 1:** Outcome criteria for the clinical and radiographic assessment of restorations and teeth

	**Control restoration**	**Hall PMCs**
**Successful**	restoration appears satisfactory, no intervention required no clinical signs or symptoms of pulpal pathology no pathology visible on radiographs tooth exfoliated	restoration appears satisfactory, no intervention required no clinical signs or symptoms of pulpal pathology no pathology visible on radiographs tooth exfoliated
**'Minor' failure**	secondary caries, or new caries clinically or radiographically restoration fracture or wear requiring intervention restoration loss; tooth restorable reversible pulpitis treated without requiring pulpotomy or extraction	crown perforation new caries (around margins) restoration loss; tooth restorable reversible pulpitis treated without requiring pulpotomy or extraction
**'Major' failure**	irreversible pulpitis or dental abscess requiring pulpotomy or extraction inter-radicular radiolucency restoration loss; tooth unrestorable internal root resorption	irreversible pulpitis or dental abscess requiring pulpotomy or extraction inter-radicular radiolucency restoration loss; tooth unrestorable internal root resorption

**Figure 6:**
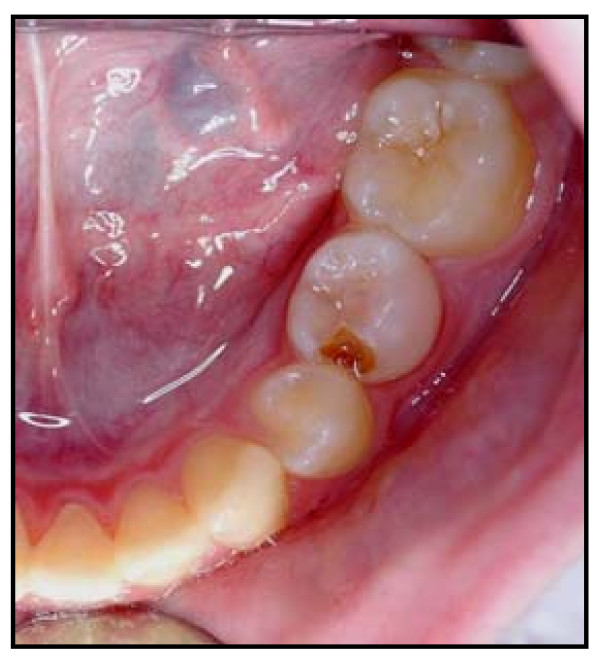
Clinical photograph of 'Minor' failure of Control restoration on tooth 75 (LLE); restoration lost and caries progression. Patient randomisation number 92.

**Figure 7:**
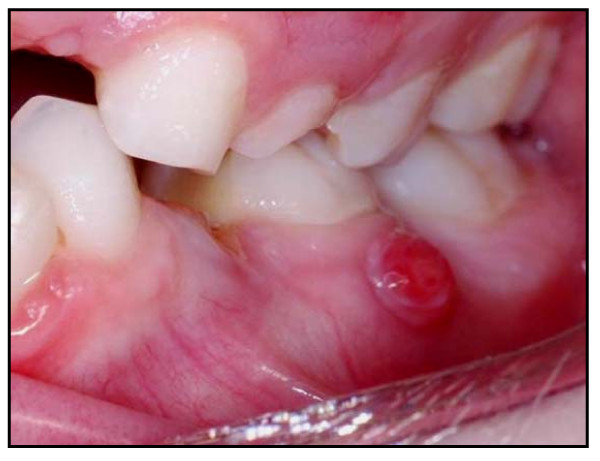
Clinical photograph of Control restoration 'Major' failure with sinus visible. Patient randomisation number 19.

• Successful;

• 'Minor' failure – restoration failure, or reversible pulpitis, which could be managed by repair or replacement of the restoration (see Figure 6); and

• 'Major' failure – signs or symptoms of irreversible pulpal damage, such as dental abscess, or tooth broken down and unrestorable (see Figure 7 for an example).

These outcome criteria were chosen on the basis that they provided pragmatic, relevant information for clinicians and patients.

Data were entered into an Access database (Microsoft XP Professional, Microsoft Corporation) and analysed using Minitab 14 (Minitab^® ^Statistical Software, Minitab Inc.).

### Radiographic assessment

Radiographs were analysed by one author (DE) using a standard illuminated radiograph viewer, screened off, in a darkened room and using two times magnification. Data recorded from the initial radiographs included:

• quality of radiographs for; exposure/developing, visibility of study tooth crown, visibility of study tooth furcation area;

• classification of the most significant carious lesion affecting the tooth as either occlusal (requiring a Class I restoration) or aproximal (requiring a Class II restoration);

• caries less than/equal to half way through dentine or greater than half way through dentine; and

• presence or absence of signs of pathology at furcation region (this criterion was only assessed if the inter-radicular area was recorded on the film, and close proximity of the successional permanent tooth did not confound the diagnosis).

For radiographs taken at recall visits, additional criteria included:

• whether the fit of the PMC was satisfactory (crown margin through both mesial and distal contact points);

• presence or absence of a satisfactory Control restoration; and

• any evidence of caries progression or of new carious lesions.

Investigation of inter- and intra-examiner reproducibility and repeatability were carried out using a computer generated randomisation table to select 10% of individual radiographs, which were then reassessed by two of the authors (DE and NI). Kappa analysis values ranged from 0.60 to 0.84 ('good' to 'very good').

### Data analysis

Data gathered from all proformas were entered into an Access database (Microsoft XP Professional, Microsoft Corporation) and analysed using Minitab 14 (Minitab^® ^Statistical Software, Minitab Inc.). Chi square test was used to analyse data on:

• relationship between the extent and position of the initial carious lesion and the occurrences of the outcomes of pain episodes, 'Major' failure and 'Minor' failure;

• failure of Class I GIC restorations compared with other restorations and failure of Class II GIC restorations compared with other restorations.

• patient, parent and child preferences for the control or Hall restoration; and

• patient preferences and the influence of whether the control or Hall restoration was carried out first or second.

McNemar's test was used to analyse paired data on:

• pain episodes, 'Major' failures and 'Minor' failures in the control group and the intervention group.

## Results

### Dentists

**Figure 8:**
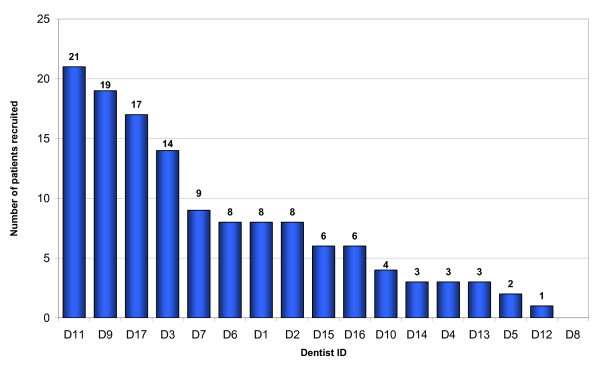
Patient recruitment pattern by individual GDP (n = 132 patients).

17 dentists recruited 132 patients over a period of two years six months (July 2001 to January 2004). Each dentist recruited between 0 and 21 patients, with the distribution shown in Figure 8.

### Patients

**Figure 9:**
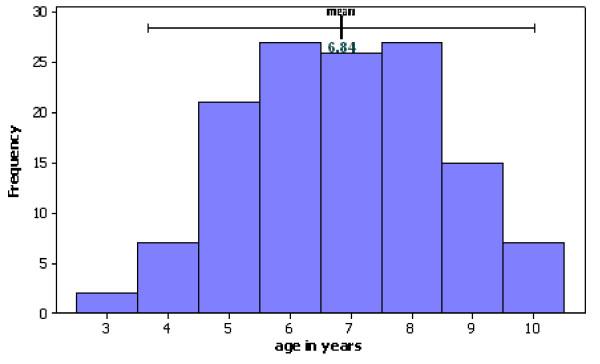
Histogram of age of children on entry to trial (n = 132 children).

69 males and 63 females were recruited from three to 10 years of age (mean 6.8 years; SD 1.58) with the distribution shown in Figure 9. Two children were three years of age and six children were 10 years of age. In the inclusion criteria, the lower age of four years was originally chosen because it was felt that children any younger would not tolerate radiographic examination. This decision was not based on clinical rationale relating to the treatments. It was, therefore, decided to include these patients. With the patients over nine years of age, the recruiting GDPs anticipated that the teeth were still likely to be present after two years. As the study was a split mouth design, inclusion of these teeth would not bias either for or against the intervention or control and they were, therefore, also included in the analyses.

### Study teeth

132 matched pairs of teeth were entered into the study with the distribution being evenly spread by tooth type (first or second primary molar) and arch (maxillary or mandibular), and is shown in Table 2. Seventy three study teeth pairs (55%) were first primary molars and 59 (45%) were second primary molars.

**Table 2:** Distribution of teeth entered into trial.

**Tooth type**	**No of teeth**	**% of total**
Maxillary first primary molar	62	**23**
Mandibular first primary molar	84	**32**
Maxillary second primary molar	66	**25**
Mandibular second primary molar	52	**20**
Total	**264**	**100**

### Characteristics of initial caries lesions

**Figure 10:**
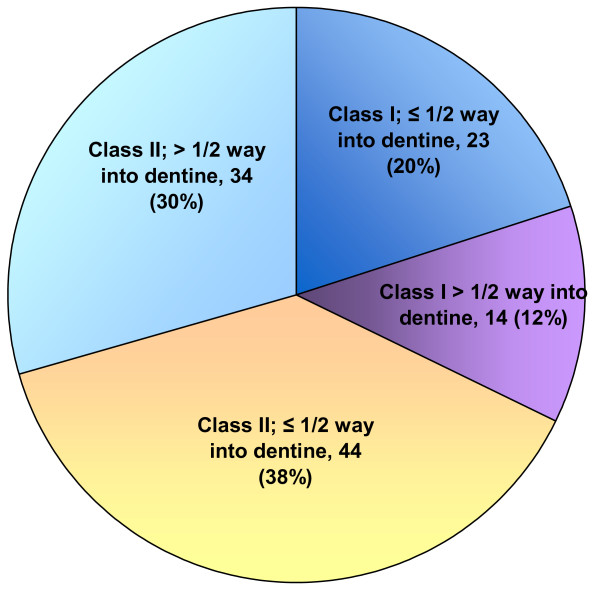
Mean values for distribution of caries lesions for study teeth (n = 229).

There was no radiographic information for 56 teeth out of the 264 entered into the trial (31 had no radiographs available; radiograph quality too poor for assessment for 10 teeth; lesion too minimal for site to be confirmed for 15 teeth). However, for 21 of these 56 teeth with no radiographic information on the initial lesion, the GDPs dental chart and the location and extent of subsequent restorations viewed radiographically were used to add information regarding initial lesion characteristics. Therefore, data regarding site and extent of the initial lesion were available for 229 of the 264 teeth entered into the trial (86%). This comprised 115 Hall teeth and 114 control teeth, with similar distributions for location and extent of caries lesions (Figure 10). A mean of 58% of lesions extended ≤ 1/2 way into dentine and 42% >1/2 way into dentine, with 32% of the lesions on the occlusal surface and 68% on an aproximal surface.

### Provision of treatments – Control restorations

Control restorations were placed in 128 teeth out of the 132 allocated to the control group (97%). For the four teeth where no restoration was placed, three children were unable/unwilling to receive any treatment and for one patient, no reason was given. Table 3 details whether complete or incomplete caries removal was carried out and the type of restoration provided. 103 of the 132 teeth in the sample (78%) were recorded as having complete caries removal carried out. The spread of restorative materials chosen by the dentists comprised: glass ionomer cement (69%); amalgam (8%); compomer (5%); composite (11%); PMC (1%); and fissure sealant (2%). Four teeth (3%) had no restoration provided. The one PMC that was placed was fitted in error as there was confusion around the randomisation process. The dentist prepared the tooth before fitting the PMC, although no caries removal took place. The reasons for incomplete caries removal in the Seventy three study (22%) scheduled for Control restorations are shown in Table 4. An air rotor handpiece was used for cavity preparation in 114 out of the 128 teeth (89%) where some treatment was carried out.

**Table 3:** Materials and caries removal for Control restorations.

**Material used to restore teeth**	**Number of teeth where caries removal was complete**	**Number of teeth where caries removal was incomplete**		**Total**
			
		***partial caries removal***	***no caries removal***	
Glass ionomer	71	20	0	**91**
Amalgam	11	1	0	**12**
Compomer	7	1	0	**8**
Composite	14	0	0	**14**
PMC (some prep of contact points)	0	0	1	**1**
Fissure sealant	-	-	2	**2**
No restoration provided	0	0	4	**4**
***Total for subgroups***	*103*	*22*	*7*	**132**

**Total**	**103**	**29**		**132**

**Table 4:** Reasons recorded by GDP for incomplete caries removal during provision of Control restoration.

**Reason for incomplete caries removal**	**Number**
Lack of cooperation/child becoming distressed	17
To avoid pulpal exposure	3
Under enamel, sealed in	2
No local analgesia used	2
No reason given	5
**Total**	**29**

### Provision of treatments – Hall PMC

PMCs were placed using the Hall Technique on 128 out of 132 teeth (97%) allocated to the intervention arm, with the fit being assessed by the GDPs as satisfactory for 124 of the 128 teeth (97%). All four teeth where no Hall PMC could be fitted were maxillary first primary molars and the reasons for a PMC not being placed are shown in Table 5. The four cases where a PMC was not fitted and the four cases where no restoration was placed involved a total of six patients. In two patients, neither treatment could be provided.

**Figure 11:**
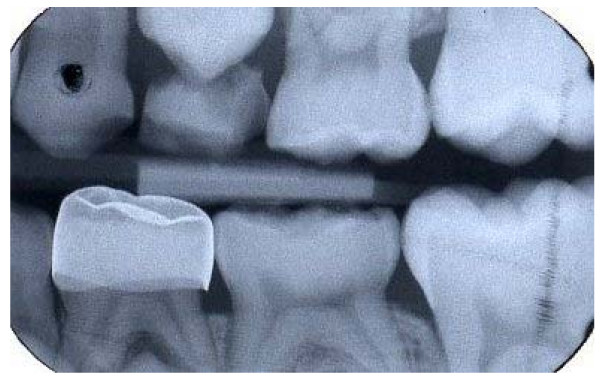
Radiograph of Hall PMC on tooth 74 (LLD) recorded as satisfactory fit. Patient randomisation number 7.

**Figure 12:**
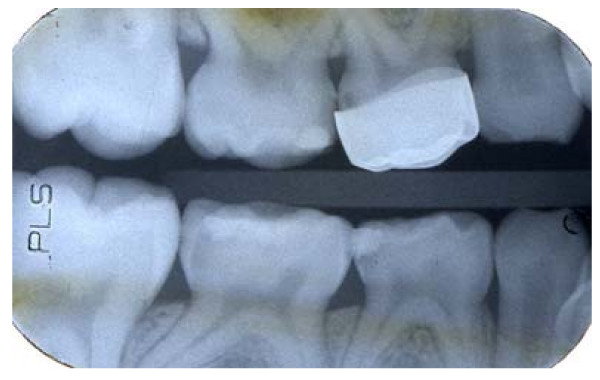
Radiograph of Hall PMC on tooth 54 (URD) recorded as unsatisfactory fit. Patient randomisation number 34.

Of the 128 teeth where Hall PMCs were fitted, 119 (93%) had at least one set of bitewing radiographs taken at follow-up appointments, and from which the radiographic appearance of the Hall PMC fit could be judged. Radiographs were not available for nine teeth where Hall PMCs had been successfully fitted as two patients did not attend any follow-up appointments, one tooth exfoliated before the next follow-up appointment where radiographs would have been taken, and six teeth had no follow-up radiographs for unknown reasons. The fit of the Hall PMC was judged from the radiographs to be unsatisfactory if one or both proximal margins of the Hall PMC were not cervical to the contact points (see Figure 11 and 12), and this was the case for 18 of the 119 (15%) teeth. Seventeen out of 18 of the teeth were first primary molars. All four PMCs assessed as being an unsatisfactory fit by the GDPs were included within the 18 PMCs assessed by the authors (DE and NI) as being an unsatisfactory fit radiographically.

**Table 5:** Teeth where Hall PMC was unable to be fitted and reason recorded by GDP.

**Reason recorded why crown not fitted**	**Tooth**	**Number**
"extra cusp on maxillary Ds, couldn't get crown to fit without major adjustments"	64	**1**
"patient unable to cooperate, very nervous"	64	**1**
"couldn't get crown to fit"	54	**1**
"all (crowns) were too big"	54	**1**
**Total**		**4**

### Use of separators prior to fitting Hall PMCs

Orthodontic separators were used for 17 cases out of the 128 intervention teeth (13%) where PMCs were successfully fitted, but their use was not evenly distributed amongst GDPs in the trial, with only seven out of the 16 GDPs who fitted Hall PMCs using them. Four of the dentists who used separators did so for only one case; one dentist used them for six out of their eight cases, with the final dentist using them for seven out of the eight cases they enrolled in the trial. Table 6 shows the relationship between use of separators and whether the Hall PMC was assessed as fitting satisfactorily or not. A Chi square test showed no evidence of association (p = 0.810) between use of separators and satisfactory Hall PMC fit.

**Table 6:** Use of separators and subsequent satisfactory fit of Hall PMC where a PMC was fitted (n = 128 teeth).

	**Hall PMC fit satisfactory**	**Hall PMC fit not satisfactory**	**Total**
Separator used	15	2	17
Separator not used	95	16	111
Total	**110**	**18**	**128**

### Subjective assessment of discomfort, if any, experienced by the child

For 89% of Hall PMCs, procedures were rated by the GDPs as causing "no apparent discomfort" to "mild, not significant", while for Control restorations, the figure was 78%, and the difference was statistically significant (Chi square test, p = 0.012). The distribution of this data is shown in Figure 4.

### Preference for either procedure

**Figure 13:**
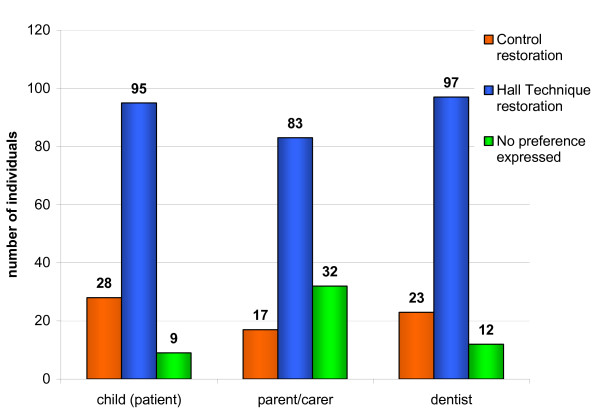
Patient/carer/dentist treatment preference (n = 396 for 132 treatment events).

Following completion of both procedures, the dentists recorded which procedure (Hall PMC or Control restoration) they, the child and their carers had preferred. Over nine out of 10 children and dentists, and three-quarters of carers, expressed a treatment preference (Figure 13). For 77% of the children, 83% of carers and 81% of dentists, the preference was for the Hall PMC, and this was statistically significant (one sample Chi square goodness of fit test; P < 0.0001) for all three groups. Therefore, the null hypothesis that children, carers and dentists would not have a preference was rejected.

The use of separators did not influence either the level of discomfort experienced by the child when having a Hall PMC fitted, as assessed by their dentist, or the preference for either technique expressed by the child, their parent or their dentist. When the Control restoration and Hall PMC were provided during the same appointment, the children's preferences were not dependent on which restoration was carried out first (Chi square analysis; P = 0.203).

### Consequences of increasing the occlusal vertical dimension

**Figure 14:**
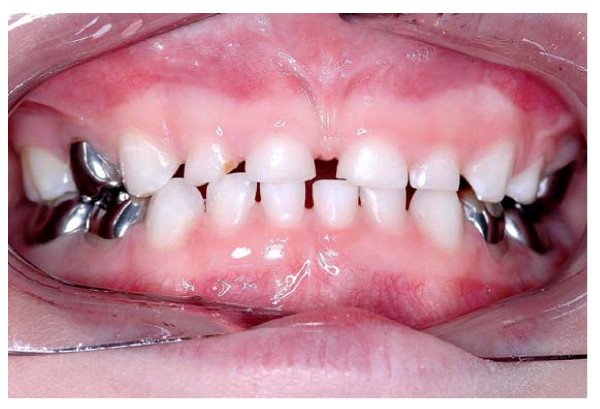
Clinical photograph of a patient with six PMCs fitted using the Hall Technique at separate appointments. The occlusion has adjusted to give even contact between the arches.

As the Hall Technique does not involve any occlusal reduction of teeth, it is inevitable that the placing of a Hall PMC will increase the occlusal vertical dimension (OVD). GDPs measured this increase at the incisors immediately after placement of the Hall PMC. The mean reported value for all teeth was 2.4 mm (SD 0.13, range 0–4 mm). For first primary molars this was 2.3 mm and for second primary molars the mean value was a little higher at 2.5 mm. Even occlusal contact was recorded on both sides of the arch for all 129 children at the one year recall appointment. An example of a patient treated with Hall PMCs demonstrating re-established even occlusal contact is shown in Figure 14.

### History of TMJ pain

There was no history of TMJ pain, and no difficulty with eating reported by either the child or their parent for all 129 children assessed at one year recall.

### Time taken for placement of restoration

For 84 patients (64%), the treatments took place at the same appointments and for 48 (36%), at separate appointments. The time taken to explain and complete each procedure was reported for 129 Hall PMC procedures and 128 Control restoration procedures. For the Control restorations, a mean time of 11.3 minutes (range 4 to 32 minutes; SD 5.5) was reported, with a mean time of 12.2 minutes (range 2 to 40 minutes; SD 8.3) for the Hall PMCs. Table 7 shows a breakdown of the times for each procedure.

**Table 7:** Time taken (in minutes) for explanation and treatment for Control restoration and Hall Technique.

**Treatment type**	**Mean explanation time (mins)**	**Mean treatment time (mins)**	**Mean total time (mins)**
Control restoration	2.8	8.5	**11.3**
Hall technique	**3.9**	**8.3**	**12.2**

### Follow-up period

The minimum period of 23 months (rather than 24 months) was chosen to take account of minor variation in dental practices arranging yearly recalls. Data were analysed for all patients with follow-up data at a minimum of 23 months (mean 28 months), for the period 0 – 36 months following enrolment into the trial. Patients with data only at less than 23 months were not included in the follow-up data analyses. Of the 132 patients enrolled in the study, there were 124 patients (94% follow-up rate) who met these criteria. Table 1 details the outcome criteria for the clinical and radiographic assessment of the restorations and teeth.

### Radiographic assessment of outcomes

For the period of 0 to 36 months, there were a total of 422 follow-up radiographs (211 Control and 211 Hall) available for the 124 patients meeting the inclusion criteria, as there was more than one set of radiographs for some patients. There was radiographic information for 117 patients out of 124 (94%) where this might have influenced the assessment of success or failure (one patient's teeth exfoliated before the one year recall and six had no follow-up radiographs for unknown reasons). The results for radiograph quality assessment are shown in Table 8. The presence or absence of new caries or caries progression could be determined (codes 0 and 1 for exposure/developing; codes 0 and 1 for visibility of study tooth crown) for 114 Control restoration allocated teeth (88%) and 117 Hall PMC teeth (91%). Information on the presence or absence of inter-radicular radiolucencies (codes 0 and 1 for exposure/developing; codes 0 and 1 for furcation visibility) could be determined for 110 teeth allocated to Control restorations (85%) and 81 Hall PMC teeth (63%). The reduction in visibility of the furcation area for Hall PMC teeth compared with the Control restoration teeth can be explained as the PMCs are fitted with no occlusal reduction, and so lengthen crown height, reducing the area of film adjacent to the furcation area.

**Table 8:** Quality assessment of follow-up radiographs for patients with minimum follow-up of 23 months; range of radiographs 0–36 months.

			**Follow-up radiographs**			
			
**Category**	**Criteria**		**Control restoration**		**Hall PMC**	
			
			**number**	**%**	**number**	**%**
**Exposure/developing**	good contrast between enamel/dentine	**0**	165	78	180	85
	poor contrast, but useable	**1**	40	19	26	12
	only useable to confirm presence or absence of teeth or PMC	**2**	5	2	5	2
	teeth not visible/unusable	**9**	1	1	0	0

**Total**			**211**		**211**	

**Visibility of study tooth crown**	whole crown visible, no overlaps	**0**	133	63	188	89
	overlaps confined to enamel	**1**	58	28	7	3
	significant overlap	**2**	3	1	1	1
	carious lesion visible, but part of crown off-film	**3**	14	7	14	7
	crown not visible/unusable	**9**	3	1	1	1

**Total**			**211**		**211**	

**Visibility of study tooth furcation area**	furcation area visible	**0**	174	83	133	63
	furcation area partially visible	**1**	12	6	14	7
	furcation not visible/not usable	**9**	25	12	64	30

**Total**			**211**		**211**	

### 'Major' failures

**Figure 15:**
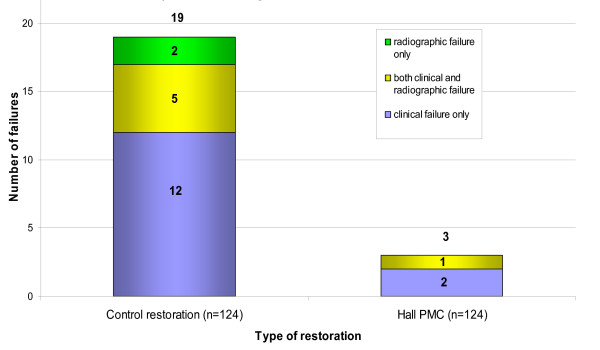
'Major' failures for Control restorations and Hall PMCs noted clinically, radiographically or both. Minimum patient follow-up 23 months; range of restoration failure 0–36 months.

There were 19 Control restoration failures ascribed as 'Major' (mean time to first failure – 19.8 months; range – one to 35 months; median – 21 months) and three for Hall PMCs (mean time to first failure – 17 months; range – three to 31 months; median – 17 months). A breakdown of whether these were noted clinically, radiographically or both is summarised in Figure 15, and Table 9 describes the failed restorations and their distribution within matched pairs. Details of the type of 'Major' failures suffered by both the Control restoration and Hall PMC teeth are shown in Table 10. McNemar's test demonstrated a statistically significant difference for 'Major' failures in favour of the intervention (Hall PMCs) (χ^2 ^= 11.2500; P < 0.000). This allows the null hypothesis of no difference in pulpal health to be rejected. The relative risk ratio of a tooth restored with a Hall crown having a 'Major' failure was 0.16 of that of a tooth restored conventionally, and gave a numbers-needed-to-treat value of 8 (95% CI = 5 – 17). For the Control restorations, although seven 'Major' failures were detected on radiographs, for only two of these was the 'Major' failure detected purely radiographically, with no record of clinical signs or symptoms at all. Only one of the 'Major' failures for the Hall PMCs was detected radiographically and this was also noted clinically.

**Table 9:** 'Major' failures of Control and Hall restorations and their distribution between split mouth pairs.

	**Control restoration**			
**Hall PMC**		*no 'Major' clinical failure*	*'Major' clinical failure*	**Total**
	
	*no 'Major' clinical failure*	103	18	**121**
	*'Major' clinical failure*	2	1	**3**
	**Total**	**105**	**19**	**124**

**Table 10:** Reasons for 'Major' failures being recorded. Minimum patient follow-up 23 months; range of restoration failures 0–36 months.

	**Criteria**	**Control restoration (Control restoration)**	**Hall PMC**
**Clinical failure**	Irreversible pulpitis	3	1
	Dental abscess, requiring pulpotomy or extraction	12	2
	Restoration loss; tooth unrestorable	2	0
**Radiographic failure with no clinical signs**	Pulp therapy carried out, but no record in notes	1	0
	Internal resorption	1	0
**Total**		**19**	**3**

The characteristics and distribution of the initial carious lesions and their relationship to teeth with 'Major' failures are shown in Table 11 and 12. The small numbers of 'Major' failures of the Hall PMCs meant it was not possible to statistically investigate any relationship between the initial lesion characteristics and failure. Chi Square analyses were undertaken to investigate any relationship between a 'Major' failure of the Control restoration and;

**Table 11:** Number of teeth with 'Major' failure or no 'Major' failure and location of initial caries lesion. Minimum patient follow-up 23 months; range of restoration failures 0–36 months.

**Location of initial caries lesion**	**Control restoration no 'Major' failure**	**Control restoration 'Major' failure**	**Hall PMC no 'Major' failure**	**Hall PMC 'Major' failure**
Caries lesion on occlusal surface	31	5	37	1
Caries lesion on aproximal surface	58	12	68	2
Location unknown	16	2	16	0
***Subtotal***	*105*	*19*	*121*	*3*
**Total**	**124**		**124**	

**Table 12:** Number of teeth with 'Major' failure or no 'Major' failure and extent of initial caries lesion. Minimum patient follow-up 23 months; range of restoration failures 0–36 months.

**Extent of initial caries lesion**	**Control restoration no 'Major' failure**	**Control restoration 'Major' failure**	**Hall PMC no 'Major' failure**	**Hall PMC 'Major' failure**
Caries ≤ 1/2 way through dentine	59	6	60	1
Caries > 1/2 way through dentine	30	11	45	2
Extent unknown	16	2	16	0
***Subtotal***	*105*	*19*	*121*	*3*
**Total**	**124**		**124**	

• occlusal or aproximal caries lesion (resulting in a Class I or Class II restoration);

• caries initially being ≤ or > 1/2 way through dentine; and

• partial or complete caries removal.

No relationship was found for the Control restoration 'Major' failure and the surface of the tooth affected by the initial lesion (χ^2 ^= 0.482; P = 0.786). There was a statistically significant relationship between a 'Major' failure of the Control restoration and an initial lesion extending > 1/2 way into dentine (χ^2 ^= 6.289; P = 0.043). For the Control restorations there was no statistically significant increase in 'Major' failures (χ^2 ^= 2.611; P = 0.106) if only partial caries removal took place compared to complete caries removal (Table 3).

### Episodes of pain

**Figure 16:**
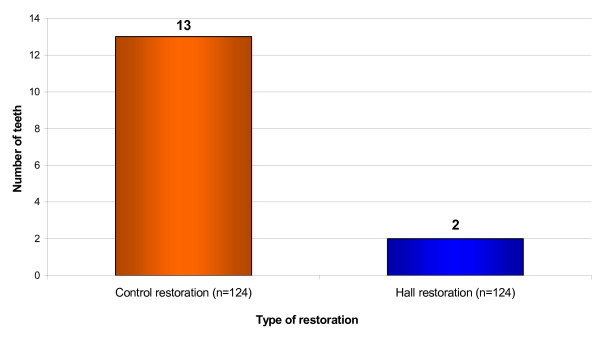
Episodes of pain from teeth treated with Control restorations and Hall PMCs. Minimum patient follow-up 23 months; range of pain episodes 0–36 months.

There were 15 recorded episodes of pain in 13 children, related to study teeth. Twelve children reported pain from a tooth that had a Control restoration placed, with one of the children having two episodes of pain. Two children experienced pain from the Hall PMC tooth, with one of these children having pain from both the Control and the Hall PMC tooth (Figure 16). McNemar's test showed a significant difference in pain episodes experienced by a child for Control and Hall restorations (χ^2 ^= 7.692; P = 0.0055) with Hall PMCs giving less pain. The relative risk ratio of a Hall tooth giving pain was 0.15 compared with a conventionally restored tooth. This gave a numbers-needed-to-treat value of 12 (95% CI 7–33).

### 'Minor' failures

**Figure 17:**
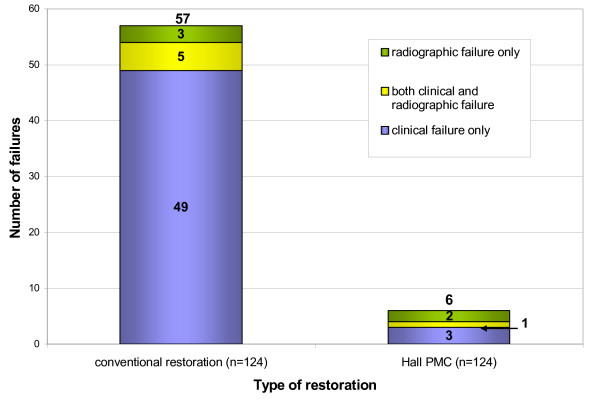
'Minor' failures for Control restorations and Hall PMCs noted clinically, radiographically or both. Minimum patient follow-up 23 months; range of restoration failures 0–36 months.

There were 57 Control restoration failures ascribed as 'Minor' (mean time to first failure -17.2 months; range – five to 35 months; median – 15 months) and six Hall PMCs (mean time to first failure – 18 months; range – six to 29 months; median – 18 months). Figure 17 summarises the 'Minor' failure data, and the types of failures for both Control restorations and Hall PMCs are shown in Table 13. McNemar's test demonstrated a statistically significant difference in 'Minor' failures (χ^2 ^= 45.45; P = 0.000) in favour of the intervention (Hall PMCs) allowing the null hypothesis of no difference in longevity of the Control restorations and Hall PMCs to be rejected. The relative risk ratio of a tooth with a Hall PMC suffering a 'Minor' failure was 0.09 compared to a conventionally restored tooth. This gave a numbers-needed-to-treat value of three (95% CI 2 to 3).

**Table 13:** Categories of 'Minor' failures for Control restorations and Hall PMCs. Minimum patient follow-up 23 months; range of restoration failures 0–36 months.

	**Control restoration**		**Hall PMC**	
	*Criteria*	*Number of teeth*	*Criteria*	*Number of teeth*

**Clinical and radiographic 'Minor' failure**	Filling lost; tooth restorable	26	PMC lost; tooth restorable	1
	Secondary caries or new caries	23	Secondary caries or new caries	1*
	Filling worn	5	Hall PMC worn through	1
			Impacted first permanent molar	1
**Radiographic 'Minor' failure alone**	caries progression visible on radiographs	3	caries progression visible on radiographs	2**
**Total**		**57**		**6**

*crown not fitted, secondary caries around restoration (followed up under Intention to treat)

** both crowns recorded as being radiographically poor fit

Table 14 details the numbers of Class I and Class II restorations and the materials used, where these were known and fitted the follow-up criteria. There was no statistically significant relationship between the use of glass ionomer for restoration of a Class I lesion and the risk of a 'Minor' failure (χ^2 ^= 1.208, P = 0.272). However, there was a statistically significant relationship for the use of glass ionomer to restore Class II lesions and a 'Minor' failure (χ^2 ^= 5.598, P = 0.018).

**Table 14:** 'Minor' failures and choice of restorative material for Class I and Class II restorations, minimum patient follow-up 23 months; restoration range 0–36 months.

	**Class I glass ionomer restoration**	**Class I other restorative material**	**Class II glass ionomer restoration**	**Class II other restorative material**
'Minor' failure	13	5	28	5
No 'Minor' failure	11	9	29	19
**Total**	**24**	**14**	**56**	**22**

## Discussion

### Basing the clinical trial in Primary Care

Restorative care alone will not solve the UK childhood dental caries problem, but for it to be even a part of the solution, it must be effective both in terms of managing the disease, and in its acceptability to children, their carers and dentists. The evidence that restorative care of primary teeth can be effective has, in many cases, been obtained from clinical trials run in Secondary Care or specialist practice settings, and it may be unrealistic to expect these findings to be generalisable to the general dental practice setting [4,18]. To support the generalisability of the outcomes from this clinical trial, the setting was general dental practice, where the vast majority of children's dentistry in the UK is carried out. Clinical trials can be run successfully in Primary Care [34], although the difficulties of doing so are widely acknowledged [35-37]. However, the very high two year patient follow-up of 94% and the commitment in data gathering shown by the GDPs involved in this study are evidence of the possibilities of Primary Care based research.

In this trial, the study co-ordinator (NI) was also a GDP at the time of patient recruitment, and this seemed to aid recruitment through motivation of other GDPs in the study [38]. The wide variation in patient recruitment by individual GDPs, with only four of the 18 GDPs meeting or exceeding their recruitment targets, mirrors the finding of the pilot study [26]. Whether this was because, as is often the case with a new technique, it suited some GDPs clinical practice more than others, or whether this was related to barriers in the general practice environment to participating in research, is uncertain.

### Preference for the restorative techniques

Children can find restorative care of carious primary molars difficult to accept. It is encouraging, therefore, that in 128 cases (97%) it was possible to fit a PMC using the Hall Technique, although in 15% of cases the Hall PMC was judged not to be fully seated. There was only one patient where it was not possible to fit a Hall PMC because of the child's inability to accept treatment, and this child also declined the Control restoration. All four teeth where no Hall PMC could be fitted were maxillary first primary molars, and 17 of the 18 cases where the PMC fit was judged radiographically as unsatisfactory were also first primary molars. Dentists using the technique report that mesial migration of second primary molars following loss of the distal marginal ridge of first primary molars can make seating a Hall PMC on these teeth problematic. Orthodontic separators can ease the fitting of a Hall PMC in such situations, although their use requires a second appointment several days later to remove the separator before fitting the Hall PMC. However, separators were only used by a minority of dentists in the study (seven out of 18 GDPs) and in only 17 (13%) of cases. The use of separators was independent of any expressed preference for either restorative technique by child or GDP, and was spread through all tooth types and both arches. Only one GDP used separators routinely (six out of seven enrolled cases). Of the other six GDPs who placed separators, five used them in a minority of the cases where they fitted Hall PMCs. This would suggest that most GDPs chose separators only when they anticipated difficulty in fitting the Hall PMC. It is of interest that in the audit of 978 Hall PMCs fitted by Dr Hall over a 10 year period, orthodontic separators were never used [25].

Most children (98%) were also able to accept Control restorations (although for one of these children, no restoration was placed as the dentist decided to simply observe the carious lesion). No Control restoration was provided for an additional three children as they were unable to accept conventional dental treatment, although two of these did accept a Hall PMC. In 29 cases (22%), caries removal was only partial before placement of the restoration. Where a reason was given for not completely removing caries, in the majority of cases (17 out of 29) this was related to a lack of patient cooperation or the child becoming distressed. In only three out of 29 cases was caries left behind to avoid a pulpal exposure. This suggests that for conventional restorative treatment, the child's ability to cope with the procedure may be the most significant factor affecting the dentists' clinical judgement as whether to remove or leave caries before restoring the tooth.

The time taken for each restoration to be placed was also similar, at around 12 minutes, with the longer explanation time for the Hall PMC offsetting the shorter treatment time when compared with Control restorations. It should be noted, though, that for 13% of the Hall PMCs, orthodontic separators were used, which necessitated an additional visit. All the GDPs were experienced in placing Control restorations, and it is possible that with increasing experience of the Hall PMCs, their treatment time could reduce, as might their use of separators.

There was a clear difference in the dentists' subjective assessment of the level of discomfort experienced by the child with both techniques. Hall PMCs were assessed as causing "no discomfort" to "mild, not significant" in 89% of cases, while for Control restorations the figure was lower, at 78% of cases. Although the dentists did not receive any specific training in assessing the level of discomfort experienced by children, they were, for each child, directly comparing the two procedures in the same child, and in 64% of the cases both procedures were completed at the same appointment. There is perhaps some confidence in the validity of dentists judging the children's perception of treatment. Holmes and Girdler recently looked at the ability of dentists to identify anxious children subjectively. They compared this judgement against the Venham and State-Trait scores of the children's anxiety [39] and reported that dentists were able to correctly identify the vast majority of children with both low and high dental anxiety. Anecdotal evidence from the GDPs was that when discomfort was observed with Hall PMCs, it was often related to the taste of the excess glass ionomer cement extruded from the margins of the crown as the Hall PMC was seated. In addition, the preferences for each procedure that the parents, children and dentists expressed, of which a component is likely to be discomfort, supports the dentists' observations of discomfort levels experienced by the child.

Preferences for either technique were expressed by 88% of children, 91% of GDPs but only 76% of carers. The reduced figure for carers was due to them not always being present in the surgery when treatment was carried out. Where a preference was expressed, this was for the Hall Technique in the majority of cases, with 77% of children, 83% of carers and 81% of GDPs favouring the Hall Technique over Control restorations.

### Effect of fitting Hall crowns on the occlusal vertical dimension

As the Hall Technique involves no occlusal reduction before fitting the Hall PMC, the procedure is inevitably associated with a premature contact following cementation, and an increase in OVD. As would be anticipated, Hall PMCs on second primary molars caused slightly more of an increase than Hall PMCs on first primary molars. However, for all the 129 cases where data were available, GDPs recorded that an even occlusal contact had re-established at the one year recall. In the experience of Dr Hall, and the authors, the occlusion equilibrates quite rapidly, usually in a matter of weeks. Although it would have been ideal to have the dentists seeing the children two weeks after the crowns were fitted to assess the occlusion, it was not possible within this study design. Trying to construct an ideal trial design within the limits of the general dental practice setting is one of the challenges that faces Primary Care based dental research.

In this study, no child re-attended their dentist following placement of a Hall PMC with signs or symptoms of occlusal dysfunction, and no child or parent reported difficulty with eating or symptoms of TMJ dysfunction syndrome when they were directly questioned by their dentist at one year or at two year recall. Orthodontists routinely use anterior and posterior bite planes, increasing the vertical dimension significantly more than the fitting of Hall PMCs. Frequently the contact of such bite planes is on one or two teeth only. In healthy children, the presence of orthodontic appliances with bite planes does not appear to place them at increased risk of developing TMJ disorders [40]. They alter the timing of vertical dento-alveolar development on a temporary basis, with an accommodation by a temporary increase in lower face height and subsequent rapid eruption of other teeth to establish more even occlusal contacts. This increase in lower face height is itself accommodated by a slowing of vertical facial growth [41]. The Hall PMCs are acting in a similar manner to such orthodontic appliances, and similarly no untoward effects were found. A recent review of the literature by Luther [42] found no evidence to support the premise that occlusal factors, including premature occlusal interferences, lead to TMJ dysfunction syndrome. Sadowsky and BeGole [43] carried out a full clinical examination and structured questionnaire on 75 adults who had undergone orthodontic treatment with fixed appliances in both dental arches, at least 10 years previously, during adolescence. They compared the results with a matched sample of patients who had not undergone orthodontic treatment and found no difference in signs or symptoms of TMJ dysfunction syndrome between the two groups.

### Survival of restorations; 'Major' failures

As this was a clinical trial, it was not possible to examine the health of the pulp histologically, and absence of symptoms and signs of pulpal disease (as detailed in Table 1) was used as a surrogate marker for pulpal health. There were 19 'Major' failures of Control restorations (15%) after a minimum follow-up period of 23 months, compared with three 'Major' failures of Hall PMCs, and this difference was statistically significant (p < 0.000) as well as clinically significant. The low failure rate of the Hall PMCs (2%) would seem to make them a successful restoration in their own right, and this is surprising given that there was no caries removal at all from these teeth. The success of the Hall PMCs when compared with the performance of the Control restorations is perhaps the reverse of what might have been expected, without an understanding that the progression of a carious lesion may be altered by changing its environment. It is interesting to compare the findings regarding the relationship of the site, and extent, of initial caries for both Control restorations and Hall PMCs, and the subsequent development of a 'Major' failure. The recommendation in the most recent edition of a standard UK Children's Dentistry textbook is that 'restoration carried out without pulp therapy in most primary molar teeth, where proximal caries has manifest clinically with the involvement of the marginal ridge, will fail. Once the breakdown of marginal ridge is evident pulp therapy is invariably required [44]. This treatment philosophy is based on a paper by Duggal *et al. *[45] and the source for this data is an MSc thesis [46]. The thesis looked at the histopathology of the dental pulp of 79 primary molar teeth with marginal ridge breakdown (MRBD). Almost all teeth (97%) showed some histological evidence of pulpal inflammation. However, in those teeth where the extent of MRBD was less than or equal to two thirds of the marginal width, pulpal inflammation was confined to the adjacent pulp horn in all of the 28 cases reported. Even in the 51 teeth where greater than two thirds of MRBD had occurred, there was only full coronal or radicular pulp involvement in 16 teeth (31%). Although this histopathology study aimed to investigate the use of the clinical appearance of the carious lesion to predict the extent of pulpal inflammation in teeth affected by dental caries, the clinical relevance of this inflammation could not be studied, and the author states; 'However, it should be borne in mind that this investigation did not study whether the changes in the pulp were reversible or not [46]. There is no information within the literature as to when the degree of inflammation becomes clinically relevant to pulpal prognosis, or at what stage, with removal of the insult (infected tissue), the pulpal tissue has become too inflamed to recover'. The evidence from the clinical trial of the Hall Technique would certainly seem to contradict the absolute importance of marginal ridge integrity in predicting the need for pulp therapy. It is unfortunate that in the Hall trial, presence or extent of MRBD was not recorded by the GDPs as, in retrospect, this might have helped to clarify the relationship between MRBD and subsequent clinical indications of pulpal pathology. However, it is known that 30% of the 229 lesions included in the Hall PMC study, and for which initial radiographs were available, were Class II lesions with caries extending over half way into dentine. As such, it is highly likely that the majority of these would have evidence of some degree of MRBD, firstly because of the extent of the lesion, and secondly because none of the GDPs routinely took bitewing radiographs. They would, therefore, have had to clinically detect an aproximal lesion before taking radiographs with a view to entering the child into the trial. However, the site of the lesion did not affect whether or not the teeth went on to display the clinical signs and symptoms of pulpal pathology. In addition, only 12 of the 78 control teeth (15%) recorded as having aproximal lesions resulted in a 'Major' failure. Given that pulp therapy in the hands of specialists can often have a failure rate of over 10% [47-50], and that it is quite an invasive treatment for a child to be expected to cope with, it would seem prudent to revise the recommendation made by Duggal [44].

The high "Major" failure rate for the Control restorations (15%) is disappointing but is a reflection of the outcomes related to current practice in the general practice environment. The poor outcomes for conventional restorations in this study are supported by lower levels of evidence such as the retrospective analyses of GDP records [51].

Non-visualisation of the furcation area for 37% of the Hall PMC teeth could lead to an under-diagnosis of pulpal pathology. However, for the control teeth, only two out of 19 "Major" failures were detected on radiographs alone (with 15% of radiographs unable to show the furcation area clearly). So the clinical impact of being less likely to detect pathology in the furcation area is, in real terms, debatable.

### Survival of restorations; minor failures

The high minor failure rate for Control restorations (13% (n = 129) at 1 year, 46% (n = 124) at minimum 23 month follow up) was disappointing, and was much higher than failure rates generally reported in the literature. There are several possible reasons for this. Unlike the present study, many studies reported in the literature are carried out in Secondary Care settings, with operators working with specified materials on particular cavity types and sizes. As well as those variables, it is likely that a significant factor in the present study was the high number of Class II restorations (68% as assessed radiographically), the majority of which (73%) were restored with conventional glass ionomer restorative materials, which are no longer recommended for use in Class II cavities [52]. A significant relationship was found between 'Minor' failure and the use of glass ionomer for Class II cavities but there was no association between glass ionomer for Class I cavities and 'Minor' failure. Nevertheless, use of glass ionomer material reflects current practice amongst this group of GDPs, and as the study was of split-mouth design, the Hall PMCs were being fitted in the same challenging environment by the same clinicians.

There were six "Minor" failures of the Hall PMCs (Table 13). While it might be assumed that the Hall PMCs would be particularly susceptible to occlusal perforation, as they are fitted without occlusal reduction of the tooth, this was not found to be the case in this study. Perforation and decementation rates were slightly lower than those reported in the literature with PMCs fitted conventionally. A retrospective analysis of survival of PMCs placed in a specialist paediatric dental practice [16], reported that out of 1,010 PMCs reviewed at a mean time of 2.13 years, 2.1% suffered a perforation and 0.9% became decemented. This was with 55% of the PMCs being placed under general anaesthetic and 98% under rubber dam; yet both of these figures are equivalent to those found with the Hall PMCs (0.8% perforating and 0.8% decementing). Other studies report similar or higher failure rates: 2% perforation and 14% decementation [53], 4.4% perforation and 8.4% decementation [54]. Two teeth with Hall PMCs (both recorded as being a poor fit) were classified as 'Minor' failures with radiographs indicating secondary caries around the cervical margin of the tooth. Again, this figure (1.6%) is comparable to that reported in the literature – 1.2% [54]. Despite there being quite a high level of PMCs judged as being an unsatisfactory fit radiographically (15%), the very low failure rate for the PMCs over the two year period (both for "Major" and "Minor" failures) seems to indicate, with the numbers of patients in this study, this criterion was not of great clinical relevance.

### Sealing in caries

**Figure 18:**
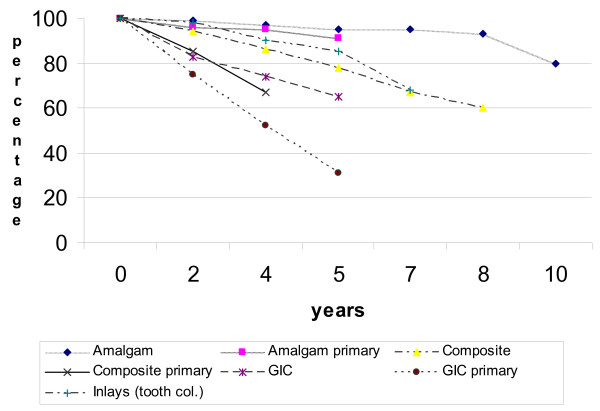
Estimates of longevity of dental restorations (in permanent and primary teeth). Reprinted with permission from Evidence Based Dentistry, Chadwick *et al*., 2002: 3; 96–99, Copyright 2002, Macmillan Publishers Ltd.

Hall PMCs were not only a more successful restorative technique for carious primary molars than the conventional techniques used by this sample of GDPs, they were also a successful restoration in their own right, achieving comparable survival rates to conventional restorations placed under more favourable conditions [55] (and see related Figure 18). This is intriguing, as there was no caries removal at all, and in 42% of cases the caries was assessed radiographically as being > 1/2 way through dentine, a stage at which it is very likely there would be some pulpal involvement [45].

Marsh's ecological plaque hypothesis [56] highlighted the very specific environment required by potentially cariogenic bacteria in order to take part in the initiation and progression of dental caries, and suggested that this was an opportunity for preventive intervention. If the environment of an actively cariogenic plaque biofilm can be altered, for example by sealing in the caries with a restoration and so isolating it from nutrients from the oral cavity, then the caries process could arrest. This study is only one of several clinical studies supporting this approach. A recent Cochrane review compared the effect of incomplete versus complete caries removal on pulpal health, and demonstrated an overall benefit to pulpal health when caries was only partially removed [28]. The findings of the current study offer further support to the concept of managing dental caries as a process that occurs in the plaque biofilm, with its consequences (lesion formation and progression) potentially arrestable by plaque control. Thus carious lesions need not be considered as irreversible disease requiring complete excision. However, caution should be exercised in applying these findings to the general management of dental caries without being aware of potential pit falls. As stated by van Amerongen *et al. *[57], with regard to arresting caries by sealing it in with a restorative material, 'the seal's the deal!'. If the seal isn't complete, then the caries can progress. It is technically demanding for even an experienced clinician to place a restoration with total confidence that there is a complete seal around the whole perimeter of the restoration (particularly for Class II restorations), and once that restoration has been placed, then the integrity of that seal must be monitored and maintained throughout life. In addition, clinicians show wide variation in simply diagnosing the presence or absence of dental caries; how reliable are they likely to be in assessing whether existing caries is arrested, or is progressing over time? And what happens when a patient with sealed-in caries changes dentists?

The evidence from this study is that Hall PMCs allow a reliable, low-maintenance seal to be achieved by GDPs. In primary teeth managed with the technique, caries can be safely left rather than removed. The use of Hall PMCs, however, is generally limited to managing carious primary molars, and even then they will not suit every carious molar, every child or every GDP. For example, the aesthetics of the crown could be a point of discussion. If the concept of sealing in some dental caries, rather than excising it, is to be applied when restoring the carious permanent dentition, then further studies, based in Primary Care, are needed to validate restorative techniques by which GDPs can reliably achieve, monitor and maintain an effective seal under practice conditions. If patients are to benefit from the smaller cavity sizes and improved pulpal health which the 'sealing-in' approach to managing dental caries promises, then this is a research challenge which restorative dentistry in the 21^st ^century must address.

## Conclusion

In summary, both Hall PMCs and Control restorations could be provided for the great majority (≥ 97%) of children in the study, and in a similar time. However, the Hall Technique was assessed by GDPs as causing significantly less discomfort compared with conventional restorative techniques, and was preferred to Control restorations by a significant majority of patients, carers and GDPs. This study supports other work indicating that the progress of dentinal caries can be significantly slowed, and perhaps even arrested, beneath a well sealed restoration. After two years, Hall PMCs were a more successful method for managing caries in primary molars than the Control restorations placed by GDPs in this high caries risk group, both for signs/symptoms of pulpal disease and longevity of the restorations. The Hall Technique appears to offer an effective treatment option for managing dental caries in primary molar teeth.  Further information can be found here :  .

## Competing interests

This clinical trial was funded in 2000 through a research training fellowship grant from the Chief Scientist Office of the Scottish Executive and financial support from 3 M/ESPE and EastRen. The sponsors of this trial had no role in its design; the collection, analysis or interpretation of data; or dissemination of results. The authors had final responsibility for the decision to submit for publication. The views expressed in this report are those of the authors and do not necessarily represent those of the funding sources.

## Authors' contributions

NI and DE conceived of the study, participated in its design, coordination, and manuscript preparation. NI carried out the study, data collection, input, and analysis. DS participated in the design of the study, provided statistical advice and assisted with statistical analysis. All authors read and approved the final manuscript.

## Pre-publication history

The pre-publication history for this paper can be accessed here:


